# LR3-LI4 needling side and configuration are associated with phase-specific cortical activation and network organization: a functional near-infrared spectroscopy study in healthy adults

**DOI:** 10.3389/fneur.2026.1815369

**Published:** 2026-08-13

**Authors:** Yan Xie, Hanrou Ouyang, Xiaodan Zhang, Xiaoru Zheng, Haoran Liang, Wenbin Fu

**Affiliations:** 1 The Second Clinical College, Guangzhou University of Chinese Medicine, Guangzhou, Guangdong, China; 2 The Fifth Clinical College of Guangzhou Medical University, Guangzhou, China; 3 Department of Acupuncture, The Second Affiliated Hospital of Guangzhou University of Chinese Medicine, Guangzhou, China

**Keywords:** acupuncture, cortical activation, dominant-side needling, functional connectivity, functional near-infrared spectroscopy, laterality, LR3-LI4

## Abstract

**Background:**

Whether dominant-side, non-dominant-side, or bilateral acupuncture produces comparable cortical activation remains unresolved. This study compared phase-specific cortical hemodynamic responses across these configurations at LR3-LI4 using functional near-infrared spectroscopy (fNIRS).

**Methods:**

In total, 53 healthy right-handed adults were randomized to dominant-side acupuncture (DS, *n* = 13), bilateral (BI, *n* = 15), non-dominant-side stimulation (NDS, *n* = 14), or sham (SH, *n* = 11). Cortical activity was measured across bilateral prefrontal, motor, and somatosensory regions during baseline, needle retention, and post-removal sessions. Functional connectivity and graph-theoretic metrics were analyzed as complementary network-level outcomes.

**Results:**

During retention, DS showed greater left dorsolateral prefrontal cortex (DLPFC) and frontopolar area oxygenated hemoglobin (FPA HbO) activation than BI (DLPFC: mean difference = 0.110, 95% CI 0.036 to 0.184, *p* = 0.005; FPA: 0.146, 95% CI 0.044 to 0.248, *p* = 0.007) and SH (DLPFC: 0.070, *p* = 0.040; FPA: 0.114, *p* = 0.034). NDS showed limited prefrontal effects but greater right S1 activation than BI (mean difference = 0.179, 95% CI 0.079 to 0.279, *p* < 0.001). BI exhibited biphasic somatosensory responses, with initial right S1 deactivation during retention (*t* = −2.944, *p* = 0.010) followed by post-removal rebound (*t* = 3.188, *p* = 0.006). Functional connectivity analysis suggested configuration-specific associative patterns: DS showed dynamic frontoparietal-somatosensory coupling, BI showed delayed frontoparietal-motor connectivity enhancement, and NDS showed delayed cross-hemispheric sensorimotor coupling. All active groups showed enhanced bilateral FPA and right DLPFC-FPA connectivity compared with SH post-removal (*F* = 3.207, *p* = 0.031; *F* = 3.189, p = 0.031). Graph analysis further indicated phase-related network reorganization, with significant effects for global efficiency (FDR *p* = 0.021) and clustering coefficient (FDR *p* = 0.024).

**Conclusion:**

In healthy right-handed adults, LR3-LI4 needling configuration was associated with distinct phase-specific cortical response patterns. DS was associated with greater retention-phase prefrontal HbO activation than BI; NDS showed a more somatosensory-dominant pattern, and BI showed attenuated retention-phase responses followed by post-removal network changes. These findings provide short-term neurophysiological evidence that the needling side may influence cortical responses, but they do not establish clinical superiority. Patient-based studies are needed to determine whether these cortical signatures predict therapeutic outcomes.

## Introduction

LR3 (Taichong) and LI4 (Hegu) acupuncture, often described as the Four Gates combination, is a classical distal acupoint pairing in acupuncture and has been used in clinical and neuroimaging studies of pain, cognitive-affective symptoms, and brain-network regulation (1). In clinical practice, both unilateral and bilateral needling are used, although many acupuncturists tend to favor bilateral stimulation because needling homologous acupoints on both sides of the body is assumed to produce broader or stronger therapeutic engagement (2). This assumption is clinically understandable, but it may not directly reflect cortical physiology. Sensory inputs from the two sides of the body interact through lateralized somatosensory representation, transcallosal inhibition, and attentional control mechanisms (3–6); therefore, bilateral acupuncture may alter cortical response organization rather than simply amplify unilateral stimulation. Similar principles have been observed in noninvasive brain-stimulation paradigms, where bilateral cortical stimulation does not uniformly outperform unilateral stimulation (7). Conversely, unilateral needling may be clinically valuable for patients with low needle tolerance, needle fear, local skin sensitivity, or a need to reduce treatment burden, particularly younger individuals requesting minimal insertion (2, 8). Clarifying whether dominant-side, non-dominant-side, and bilateral LR3-LI4 stimulation produce distinct cortical activation and connectivity patterns may therefore provide a neurophysiological basis for more individualized and patient-tolerable acupuncture prescriptions.

Across fMRI and multimodal EEG-fMRI studies, acupuncture-related responses have been reported in sensorimotor, prefrontal, limbic, salience, and default-mode related networks, and these responses vary with acupoint location, de-qi sensation, and stimulation intensity (9–12). MEG studies provide temporal and oscillatory evidence, showing manual acupuncture-related beta-band power decreases in primary somatosensory cortex and superior frontal regions during LI4 stimulation (13). These findings support the broader view that acupuncture responses depend on acupoint, stimulation intensity, needling sensation, body side, and analytic approach (2, 14). Recent functional near-infrared spectroscopy (fNIRS) work further supports the relevance of prefrontal-motor cortical connectivity during de-qi acupuncture (15), providing a methodological bridge to the present study. For LR3-LI4, the key question is therefore not only whether acupuncture changes brain activity, but whether the side of needling changes when and where the cortex responds. Functional near-infrared spectroscopy (fNIRS) is well suited to this question. It can record cortical hemodynamics during needle retention and after needle removal, while allowing a more natural acupuncture procedure than many scanner-based designs (15, 16). Prefrontal, somatosensory, motor, and premotor cortices are accessible to optical measurement and are relevant to sensory integration and top-down control (16). Within this cortical system, the dorsolateral prefrontal cortex (DLPFC) is relevant to executive control and pain modulation, whereas the FPA is positioned at the anterior prefrontal apex and is implicated in higher-order monitoring, affective appraisal, and integration of internally and externally directed information (17–19). S1 represents body-side somatosensory input, and M1/SMA-PM contribute to sensorimotor integration and post-stimulation motor-network organization (3, 4, 11). Prior fNIRS studies suggest that acupuncture can alter prefrontal and motor cortical activity (15, 20–22). Recent neuroimaging work also supports phase-dependent acupuncture effects in sensorimotor networks (23, 24). Current evidence comparing unilateral and bilateral acupuncture remains limited. Among 20 fNIRS acupuncture studies published to date, only one directly compared configurations in stroke patients (25). While several studies demonstrated bilateral cortical effects from unilateral acupuncture (11, 14, 26), none included direct bilateral comparisons or systematically examined dominant versus non-dominant-side effects.

Despite this progress, three gaps remain. First, many acupuncture neuroimaging studies compare active stimulation with sham stimulation but do not treat needling side as a main factor (9–12). Second, most analyses emphasize regional activation and give less attention to communication between regions (15). Third, post-removal responses are often treated as secondary, even though acupuncture effects may continue after the needle is removed (24). These gaps are relevant for LR3-LI4 because this prescription is commonly applied bilaterally, while its cortical effects may depend on body side, handedness, and phase (14, 23, 25). In right-handed participants, right-sided dominant needling may therefore engage left-hemisphere sensorimotor and prefrontal control systems differently from non-dominant side or bilateral needling. Conversely, bilateral needling may introduce interhemispheric interaction, redistribution, or delayed integration across hemispheres rather than simply increasing response magnitude (5, 6, 26). The present study analyzed a four-arm LR3-LI4 fNIRS experiment comparing dominant-side needling, bilateral needling, non-dominant-side needling, and sham stimulation. The study focused on baseline, needle retention after de-qi, and post-removal phases. Instead of asking whether LR3-LI4 acupuncture is active in general, we asked whether the same acupoint prescription produces different cortical responses when delivered to the dominant side, both sides, or the non-dominant side. We also tested whether regional activation findings were supported by functional connectivity and graph measures.

## Methods

### Participants

This randomized controlled study employed four parallel groups to compare cortical hemodynamic responses to different acupuncture configurations at LR3-LI4 acupoints: dominant-side acupuncture (DS, needling on the right side in right-handed individuals), bilateral acupuncture (BI), non-dominant-side stimulation (NDS, needling on the left side in right-handed individuals), and sham acupuncture (SH). The study was reported with reference to CONSORT recommendations for randomized trials and STRICTA recommendations for acupuncture interventions (27, 28).

Sample size was calculated based on pilot data (*n* = 8) showing a mean DLPFC oxygenated hemoglobin (HbO) activation difference between active and sham acupuncture of 0.085 μmol/L (SD = 0.090). Using a two-sided significance level of *α* = 0.05, power of 0.80, and anticipated effect size of d = 0.95, a minimum of 12 participants per group was required (G*Power 3.1.9.7). Accounting for 20% attrition, we aimed to recruit 15 participants per group (total *n* = 60) (29).

In total, 60 healthy right-handed volunteers aged 18–30 years were recruited between January and June 2024 through university advertisements. All participants were confirmed as right-handed using the Edinburgh Handedness Inventory (laterality quotient >40, indicating consistent right-hand preference) (30, 31). Exclusion criteria included congenital or hereditary diseases, endocrine or immune disorders, psychiatric or neurological conditions, severe cardiac/hepatic/renal diseases, psychoactive or neurological medication use within 4 weeks, pregnancy, acupuncture experience within 3 months, and inability to provide informed consent. The Ethics Committee of the Fifth Affiliated Hospital of Guangzhou Medical University approved this study (approval number: GYWY-K2025-45).

### Study design

**Figure 1:**
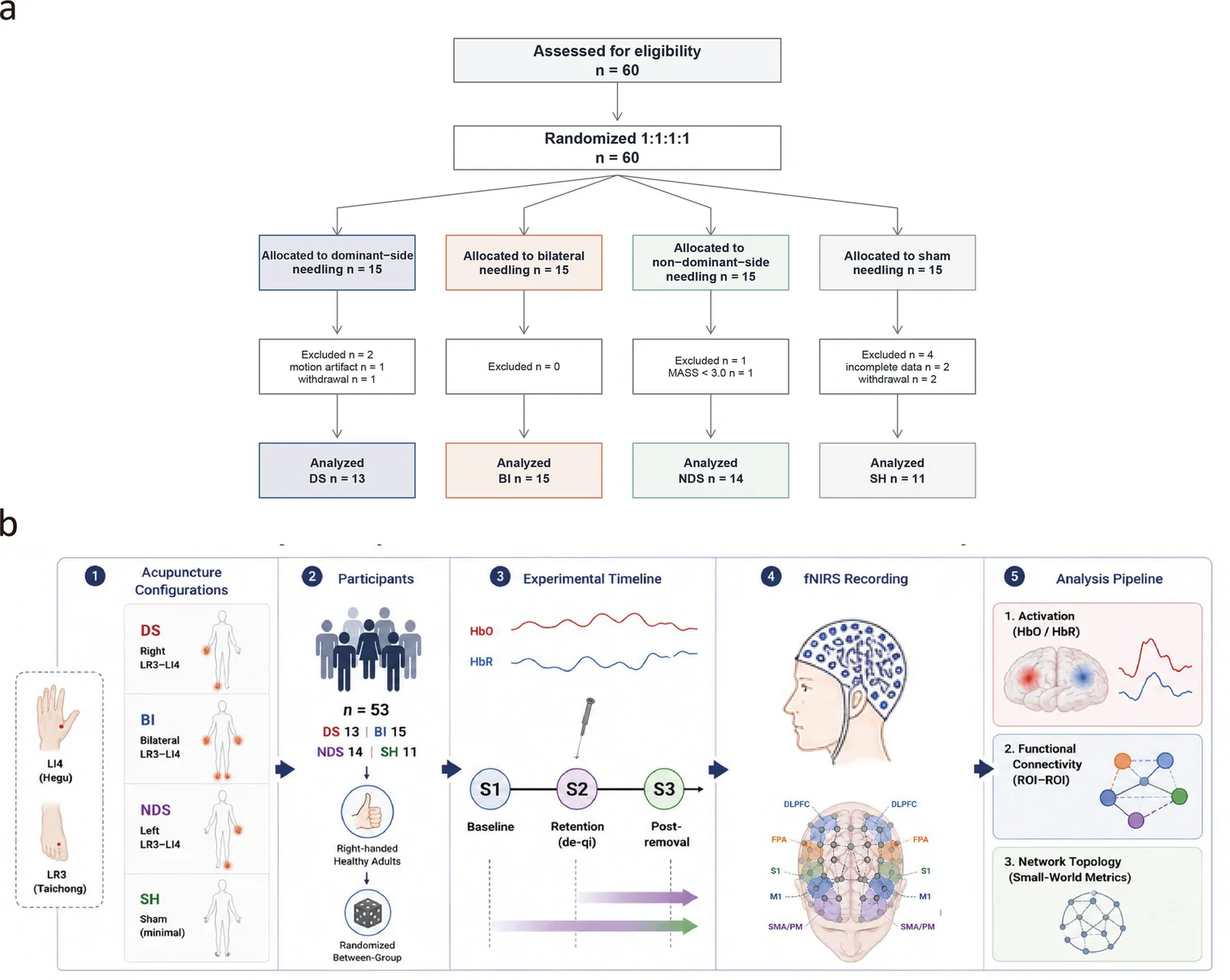
Participant flow and experimental design. **(a)** CONSORT flow diagram. **(b)** Research flow chart.

Participants were randomized to four groups using computer-generated random numbers (SPSS 26.0) in a 1:1:1:1 ratio. A researcher not involved in data collection or analysis prepared sequentially numbered, sealed, opaque envelopes containing group assignments. Envelopes were opened by the acupuncturist immediately before intervention, ensuring allocation concealment until that point. Each participant underwent three 180-s resting-state fNIRS measurement sessions: pre-acupuncture baseline (Session 1), immediately post-acupuncture during needle retention (Session 2), and post-needle removal (Session 3). Participant flow, allocation, and the overall research workflow are summarized in Figure 1.

Given the nature of acupuncture interventions, the acupuncturist performing needling could not be blinded to group assignment. Participants were informed they would receive “different forms of acupuncture” but were not told which form they received or the specific hypotheses being tested. Following each session, participants were asked, “Do you think you received real or sham acupuncture?” to assess blinding effectiveness. The fNIRS operator and data analyst were blinded to group allocation during data acquisition and initial preprocessing. Statistical analysis was conducted by a blinded statistician who received coded data (Group A, B, C, and D). Unblinding occurred only after primary analyses were completed.

### fNIRS data acquisition

**Figure 2:**
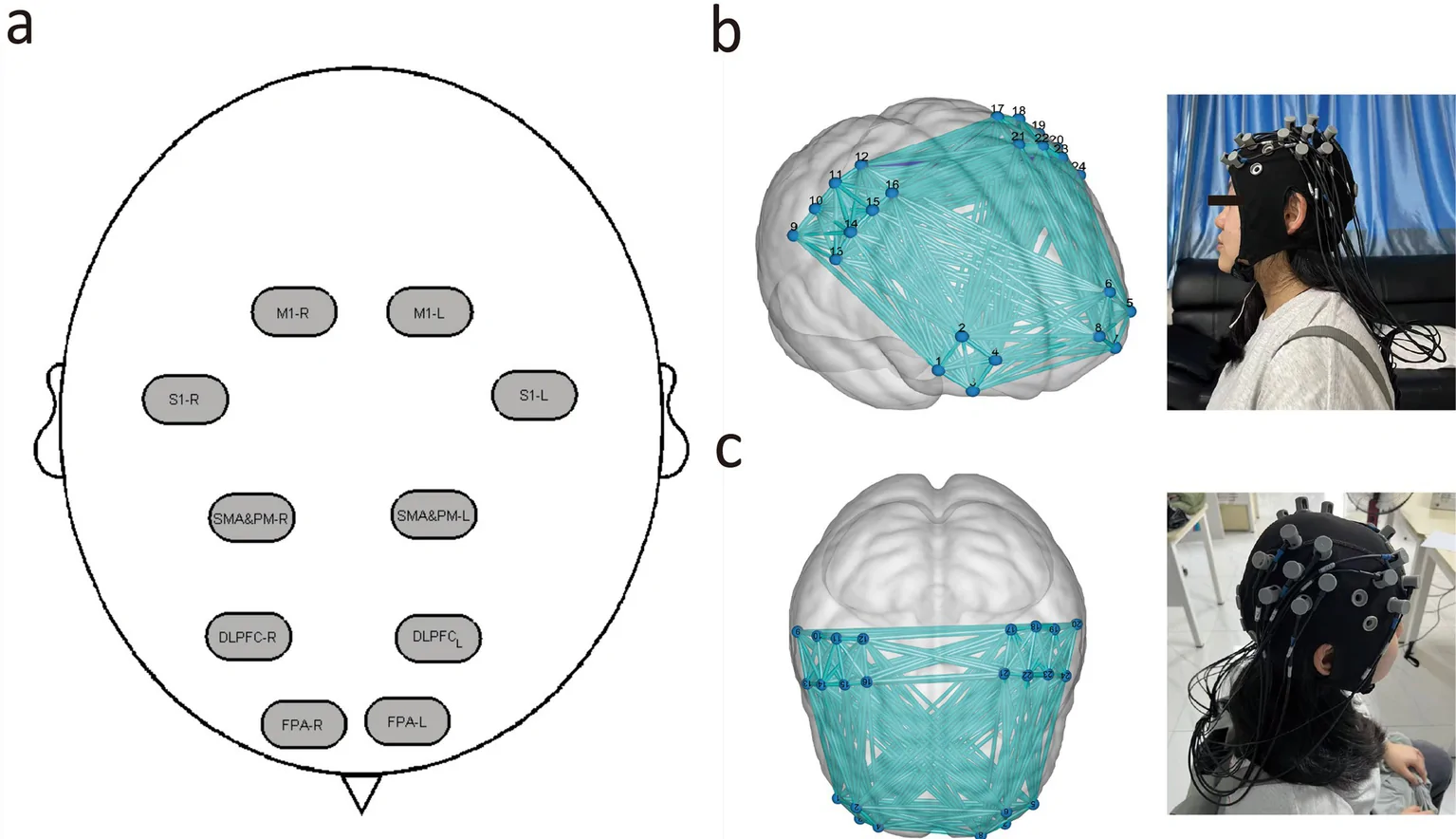
fNIRS optode placement and experimental setup. **(a)** Schematic showing bilateral measurement regions. **(b)** Lateral view of optode montage on a cortical model and participant wearing the fNIRS cap **(c)** Anterior view of the measurement array and posterior photograph of device placement. DLPFC, dorsolateral prefrontal cortex; FPA, frontopolar area; S1, primary somatosensory cortex; M1, primary motor cortex; SMA/PM, supplementary motor area and premotor cortex. The montage included 24 channels covering bilateral hemispheres.

Cortical hemodynamic activity was measured using a continuous-wave fNIRS system (NirSmart; Danyang Huichuang Medical Equipment, China) with dual wavelengths (760 nm and 850 nm) at a 11 Hz sampling frequency. The system comprised 10 detectors and 12 sources configured into 24 measurement channels with 30 mm source-detector separation, positioned according to the international 10–20 system (18). Regions of interest included bilateral dorsolateral prefrontal cortex (DLPFC), frontopolar area (FPA), primary somatosensory cortex (S1), primary motor cortex (M1), premotor cortex (PM), and supplementary motor area (SMA). Optode positions were spatially registered using a three-dimensional digitizer (FASTRAK; Polhemus, Colchester, VT, United States) integrated with NirSpace localization software (Danyang Huichuang Medical Equipment, China). Each source and detector was individually tracked to obtain three-dimensional coordinates, and channels were anatomically localized to Brodmann areas. The optode placement and participant cap configuration are shown in Figure 2.

### Acupuncture intervention

A licensed acupuncturist with over 10 years of experience performed all procedures. Two acupoints were used: Taichong (LR3), located on the dorsum of the foot in the depression distal to the first-second metatarsal junction, and Hegu (LI4), located between the first and second metacarpal bones on the dorsum of the hand. Group-specific configurations were as follows: the DS group received needling at right LR3 and right LI4, the BI group received needling at bilateral LR3 and bilateral LI4, the NDS group received needling at left LR3 and left LI4, and the SH group received non-penetrating needles at all corresponding locations bilaterally.

Participants were positioned supine in a quiet, temperature-controlled room (22–24 °C) with dimmed lighting. After skin disinfection, sterile disposable stainless steel needles (0.30 × 40 mm; Huatuo, Suzhou Medical Appliance Factory, China) were inserted perpendicularly to 15–25 mm depth for active acupuncture groups. Manual manipulation using lifting-thrusting (amplitude 3–5 mm) and rotating techniques (angle 90–180°) at approximately 1 Hz for 30 s was performed to elicit de qi sensation. De qi was defined as the composite sensation of soreness, numbness, distension, and heaviness at the acupoint and was assessed immediately after manipulation using the Massachusetts General Hospital Acupuncture Sensation Scale (MASS) (32, 33). The MASS includes 12 descriptors rated on 0–10 visual analog scales. De qi intensity was quantified as the mean score across all descriptors. Only participants reporting a mean MASS score ≥3.0 were considered to have achieved adequate de qi and were included in the retention phase. Needles were retained for 20 min following de qi achievement. The sham group received non-penetrating Streitberger needles at identical locations bilaterally (34, 35). The acupuncturist performed simulated manipulation movements at the needle handle without actual lifting-thrusting or rotation, providing tactile sensation and visual appearance of active needling while avoiding skin penetration and tissue stimulation.

### Experimental procedure

Participants abstained from alcohol, caffeine, and sedatives for 24 h before testing. Upon arrival, procedures were explained, and participants rested briefly before commencing. Participants were fitted with the fNIRS cap and positioned supine. They were instructed to remain still with eyes closed, maintain mental relaxation, and minimize movement and cognitive activity throughout testing. All measurements were conducted during resting state to eliminate task-related confounds and isolate intrinsic cortical responses to acupuncture (Table 1).

**Table 1:** Hemispheric distribution of fNIRS channels and anatomical locations.

Anatomical area	Source-detector pairs	Channel numbers
DLPFC-R (right dorsolateral prefrontal cortex)	S1-D1, S1-D2	1, 2
DLPFC-L (left dorsolateral prefrontal cortex)	S3-D4, S3-D3	6, 5
FPA-R (right frontopolar area)	S2-D1, S2-D2	3, 4
FPA-L (left frontopolar area)	S4-D4, S4-D3	8, 7
S1-R (right primary somatosensory cortex)	S7-D5	13
S1-L (left primary somatosensory cortex)	S12-D10	24
M1-R (right primary motor cortex)	S6-D7	12
M1-L (left primary motor cortex)	S9-D8	17
SMA&PM-R (right supplementary motor area and premotor cortex)	S5-D5, S5-D6, S6-D6, S7-D6, S8-D6, S8-D7	9, 10, 11, 14, 15, 16
SMA&PM-L (left supplementary motor area and premotor cortex)	S9-D9, S9-D10, S10-D8, S10-D9, S11-D9, S12-D9	18, 19, 20, 21, 22, 23

Following a 3-min adaptation phase to establish stable baseline hemodynamics, a 10-s preparation period preceded the first 180-s resting-state measurement (Session 1, pre-acupuncture baseline). Acupuncture needles were then inserted and manipulated to achieve de qi according to group assignment. After a 3-min stabilization period to minimize acute insertion effects and movement artifacts, the second 180-s measurement was conducted during needle retention (Session 2, approximately 3 min post-de qi). Needles remained in place for an additional 14 min to complete the standard 20-min clinical retention period. Following needle removal and a 3-min rest period, the final 180-s measurement assessed sustained post-acupuncture effects (Session 3). The entire session lasted approximately 35 min. Throughout the experiment, participants were monitored for adverse reactions including excessive pain, dizziness, nausea, or fainting.

### Data processing and analysis

Raw fNIRS signals were visually and procedurally inspected before hemoglobin conversion. Severe artifacts were defined as abrupt signal discontinuities, step-like baseline shifts, detector saturation, flat channels, or motion-related spikes that exceeded the local physiological range and disrupted the hemodynamic time course. Correctable motion artifacts were treated using spline interpolation in NirSpark (Danyang Huichuang Medical Equipment, China), whereas channels with persistent saturation, flat signal, or unrecoverable discontinuities after correction were excluded from ROI averaging. This correction and exclusion strategy follows standard fNIRS preprocessing recommendations for motion-contaminated continuous-wave recordings (36, 37). A 0.01–0.2 Hz finite impulse response bandpass filter was applied to reduce slow instrumental drift and high-frequency physiological noise, while preserving low-frequency hemodynamic fluctuations relevant to resting-state fNIRS connectivity and acupuncture-induced cortical responses (36, 38). Light intensity changes were converted to oxygenated hemoglobin (HbO) concentration changes using the modified Beer–Lambert law (39). Mean HbO and deoxygenated hemoglobin (HbR) changes were extracted for each 180-s session using the BlockAvg module. HbO was prespecified as the primary fNIRS endpoint because it is more sensitive to stimulation-related cortical hemodynamic changes in continuous-wave fNIRS (36). HbR was analyzed as a secondary corroborative signal to provide transparency.

Resting-state functional connectivity (FC) was assessed using Pearson correlation coefficients between HbO time series of different ROI pairs (38). FC values were computed for homotopic (interhemispheric homologous) and heterotopic (intrahemispheric and cross-regional) connections. Connectivity strength was quantified as Fisher z-transformed correlation coefficients. Channels were assigned to ROIs based on Brodmann area localization, and multiple channels within each ROI were averaged to obtain representative time series (15, 38). For graph analysis, each participant-session FC matrix was represented as a 10-node weighted network. Global efficiency, clustering coefficient, and characteristic path length were calculated to measure network integration, local clustering, and communication distance. Global efficiency reflects the capacity for distributed information integration across the network, clustering coefficient reflects local segregation or neighborhood-level grouping, and characteristic path length reflects the average communication distance between network nodes. Together, these measures provide a compact summary of the balance between network integration and local specialization (40). Group and session effects were tested using mixed-effects or repeated-measures models where appropriate. False discovery rate correction was applied within analysis families. Interpretation emphasized convergence across activation, connectivity, and graph results. Isolated uncorrected effects were not used as primary evidence.

Demographic variables were compared using one-way ANOVA (continuous variables) and chi-square tests (categorical variables). HbO concentration changes were analyzed using repeated-measures ANOVA with time (Session 1, Session 2, Session 3) as the within-subject factor and group (DS, BI, NDS, and SH) as the between-subject factor for each ROI. For ROI-level HbO comparisons, false discovery rate (FDR) correction was applied across all ROIs within each session (41). For post-hoc pairwise comparisons following significant ANOVAs, Bonferroni correction was applied. For functional connectivity analyses, FDR correction was applied across all ROI pairs within each network domain (prefrontal, sensorimotor, and front parietal). Uncorrected *p*-values are reported with correction status indicated. Paired t-tests with FDR correction compared consecutive sessions within groups. Analyses were performed using SPSS 26.0 and NirSpark software, with significance set at *p* < 0.05.

## Results

### Participant characteristics

A total of 60 healthy volunteers were initially recruited and randomly assigned to four groups (*n* = 15 per group). Seven participants were excluded before final analysis: two in the DS group (one because of incomplete fNIRS data due to excessive motion artifacts and one because of withdrawal before Session 2), none in the BI group, one in the NDS group because of inadequate de qi (MASS <3.0), and four in the SH group (two because of incomplete fNIRS data and two because of withdrawal). Thus, three participants were excluded for fNIRS data-quality or completeness reasons (DS, *n* = 1; SH, *n* = 2). The final analytic cohort included 53 participants (DS, *n* = 13; BI, *n* = 15; NDS, *n* = 14; SH, *n* = 11), and no additional participant-level exclusions were made during preprocessing. In the source-file validation workflow, all 159 retained session-level recordings from the 53 participants were processed successfully without file-level errors, and all planned ROI channel counts were retained for ROI averaging.

This resulted in final sample sizes of DS *n* = 13, BI *n* = 15, NDS *n* = 14, SH *n* = 11. Chi-square analysis confirmed no significant association between group assignment and attrition (*χ*^2^ = 4.62, *p* = 0.202). The overall sample had a mean age of 22.06 ± 3.38 years, with 75.5% women. Groups showed no significant differences in age, gender distribution, and acupuncture history (Table 2). Among active acupuncture groups, mean MASS scores were: DS 4.82 ± 1.23, BI 4.95 ± 1.18, NDS 4.67 ± 1.35. No significant between-group differences were observed (*F* = 0.342, *p* = 0.712). No serious adverse events occurred. Minor transient effects included mild needle site pain (*n* = 3), brief dizziness (*n* = 1), and minor bruising (*n* = 1), all resolving spontaneously within 24 h.

**Table 2:** Demographic and clinical characteristics by group.

Group	*n*	Age (Mean ± SD)	Gender		Acupuncture history	
			Female *n* (%)	Male *n* (%)	Ever *n* (%)	Never *n* (%)
DS	13	22.85 ± 3.52	11 (84.6%)	2 (15.4%)	12 (92.3%)	1 (7.7%)
BI	15	22.33 ± 4.12	10 (66.7%)	5 (33.3%)	15 (100.0%)	0 (0.0%)
NDS	14	21.93 ± 1.15	10 (71.4%)	4 (28.6%)	13 (92.9%)	1 (7.1%)
SH	11	21.00 ± 4.12	9 (81.8%)	2 (18.2%)	11 (100.0%)	0 (0.0%)
Total	53	22.06 ± 3.38	40 (75.5%)	13 (24.5%)	51 (96.2%)	2 (3.8%)
F/*χ*^2^ value		0.62	1.68		2.18	
*p*-value		0.61	0.64		0.54	

### Cortical activity analysis

**Figure 3:**
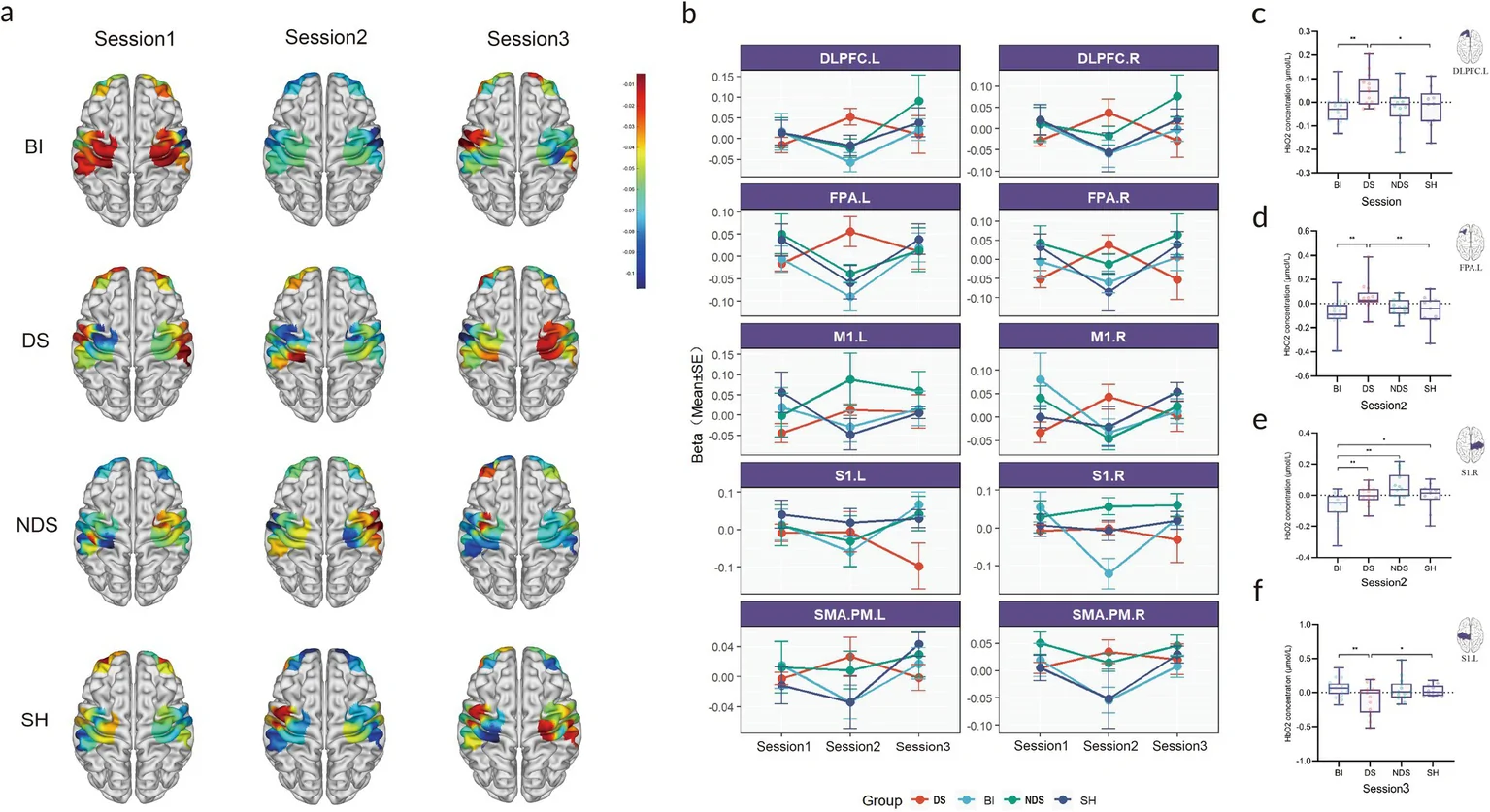
Patterns of cortical hemodynamic responses to unilateral and bilateral acupuncture at LR3-LI4 acupoints. **(a)** Cortical activation topography across intervention groups and sessions. **(b)** Temporal dynamics of regional HbO_2_ concentration changes. **(c)** Between-group comparison of left DLPFC activation during acupuncture (Session2). **(d)** Between-group comparison of left FPA activation during acupuncture (Session2). **(e)** Between-group comparison of right S1 activation during acupuncture (Session2). **(f)** Between-group comparison of right S1 activation post-acupuncture (Session3).

One-way ANOVA revealed no significant baseline differences in HbO concentration across ROIs (all *p* > 0.05), confirming successful randomization. During needle retention (Session 2), the prefrontal cortex exhibited lateralized activation patterns that differed markedly by needle configuration. In left DLPFC, the DS group showed significant activation versus baseline (*t* = 2.869, *p* = 0.014) and greater activation than SH (mean difference = 0.070, 95% CI 0.003 to 0.137, *p* = 0.040), while BI showed lower activation than DS (mean difference = −0.110, 95% CI -0.184 to −0.036, *p* = 0.005; between-group *F* = 4.164, *p* = 0.010). Right DLPFC showed activation in DS versus baseline (*t* = 2.381, *p* = 0.035) but no between-group differences (*F* = 1.850, *p* = 0.150). Left FPA demonstrated similar lateralization, with DS showing greater activation than SH (mean difference = 0.114, 95% CI 0.009 to 0.219, *p* = 0.034) and BI showing lower activation than DS (mean difference = −0.146, 95% CI -0.248 to −0.044, *p* = 0.007; between-group *F* = 4.056, *p* = 0.012). Right FPA showed DS activation versus baseline (*t* = 2.687, *p* = 0.020) and versus SH (mean difference = 0.124, 95% CI 0.006 to 0.242, *p* = 0.040), though between-group ANOVA approached significance (*F* = 2.741, *p* = 0.053). Cortical activation topography and temporal dynamics of HbO concentration changes across groups and sessions are presented in Figure 3.

Somatosensory cortex exhibited distinct hemispheric responses. Right S1 showed BI deactivation versus baseline (*t* = −2.944, *p* = 0.010) and lower activation than SH (mean difference = −0.115, 95% CI −0.216 to −0.014, *p* = 0.027), while NDS showed greater activation than BI (mean difference = 0.179, 95% CI 0.079 to 0.279, *p* < 0.001; between-group *F* = 6.679, *p* < 0.001). Left S1 showed no significant changes (*F* = 0.430, *p* = 0.733). Motor cortex showed selective NDS responses. Right M1 exhibited NDS deactivation versus baseline (*t* = −2.519, *p* = 0.026), though between-group ANOVA revealed no differences (*F* = 1.688, *p* = 0.181). The left M1 showed marginally greater activation in the NDS group compared to the SH group (mean difference = 0.135, *p* = 0.087), but between-group ANOVA revealed no significant differences (*F* = 1.799, *p* = 0.159). Neither SMA/PM region showed significant changes during retention (right *F* = 2.464, *p* = 0.073; left *F* = 1.348, *p* = 0.269).

Following needle removal (Session 3), prefrontal cortex exhibited sustained BI modulation. Left DLPFC showed BI activation increase from retention to post-removal (*t* = 2.736, *p* = 0.015), though no between-group differences emerged (*F* = 0.684, *p* = 0.566). Right DLPFC showed no significant within-group changes or between-group differences (*F* = 1.468, *p* = 0.234). Left FPA similarly showed BI activation increase (*t* = 2.480, *p* = 0.025) without between-group differences (*F* = 0.078, *p* = 0.972). Somatosensory cortex demonstrated pronounced post-removal effects. Right S1 showed BI rebound activation (t = 3.188, *p* = 0.006), reversing retention-phase deactivation, though without between-group differences (*F* = 1.109, *p* = 0.354). Left S1 showed a BI activation increase (*t* = 2.346, *p* = 0.032) and greater activation than DS (mean difference = 0.166, *p* = 0.036), while the DS group showed marginally lower activation than the SH group (mean difference = −0.128, *p* = 0.074); between-group *F* = 2.902, *p* = 0.044. Motor regions showed sustained BI activation. Right SMA/PM exhibited BI activation increase (*t* = 2.669, *p* = 0.017) without between-group differences (*F* = 0.607, *p* = 0.613). Left SMA/PM showed BI activation increase (*t* = 2.675, *p* = 0.017), while SH showed baseline-to-post-removal increase (*t* = 2.466, *p* = 0.033) without between-group differences (*F* = 0.620, *p* = 0.605). Neither right M1 (*F* = 0.566, *p* = 0.640) nor left M1 (*F* = 0.383, *p* = 0.766) showed significant within-group changes or between-group differences (Table 3).

**Table 3:** Oxy-hemoglobin (HbO) concentration (mean ± SD) by brain region and group.

ROI	Time point	DS	BI	NDS	SH	*P*
		*n* = 13	*n* = 15	*n* = 14	*n* = 11	
DLPFC. L	Session1	−0.016 ± 0.066	0.016 ± 0.181	0.014 ± 0.136	0.014 ± 0.099	0.913
	Session2	0.052 ± 0.072^*#^	−0.057 ± 0.096^*^	−0.023 ± 0.084	−0.017 ± 0.082	0.010
	Session3	0.010 ± 0.163	0.021 ± 0.107^†^	0.091 ± 0.233	0.039 ± 0.114	0.566
	Session1	−0.028 ± 0.044	0.012 ± 0.183	0.011 ± 0.148	0.021 ± 0.117	0.808
DLPFC. R	Session2	0.038 ± 0.112^*^	−0.058 ± 0.131^*^	−0.017 ± 0.083	−0.056 ± 0.153	0.150
	Session3	−0.028 ± 0.142	−0.001 ± 0.118	0.077 ± 0.183	0.022 ± 0.080	0.234
	Session1	−0.016 ± 0.064	−0.006 ± 0.121	0.050 ± 0.167	0.037 ± 0.119	0.439
FPA. L	Session2	0.056 ± 0.121^*#^	−0.090 ± 0.133^*^	−0.039 ± 0.078^*^	−0.058 ± 0.124^*^	0.012
	Session3	0.012 ± 0.146	0.020 ± 0.134^†^	0.015 ± 0.185	0.039 ± 0.115	0.972
	Session1	−0.051 ± 0.081	−0.006 ± 0.174	0.043 ± 0.162	0.033 ± 0.107	0.315
FPA. R	Session2	0.039 ± 0.085^*#^	−0.059 ± 0.118	−0.013 ± 0.098	−0.085 ± 0.164	0.053
	Session3	−0.052 ± 0.191	0.006 ± 0.147	0.065 ± 0.198	0.039 ± 0.112	0.318
	Session1	−0.044 ± 0.083	0.020 ± 0.197	−0.000 ± 0.201	0.057 ± 0.166	0.536
M1. L	Session2	0.014 ± 0.050	−0.028 ± 0.162	0.088 ± 0.242	−0.048 ± 0.128	0.159
	Session3	0.009 ± 0.150	0.016 ± 0.176	0.061 ± 0.175	0.006 ± 0.048	0.766
	Session1	−0.033 ± 0.081	0.080 ± 0.231	0.041 ± 0.093	0.002 ± 0.074	0.196
M1. R	Session2	0.043 ± 0.094^*^	−0.032 ± 0.116^*^	−0.046 ± 0.092^*^	−0.020 ± 0.141	0.181
	Session3	0.002 ± 0.118	0.013 ± 0.109	0.024 ± 0.099	0.054 ± 0.069	0.640
	Session1	−0.008 ± 0.085	0.014 ± 0.170	0.011 ± 0.203	0.041 ± 0.123	0.894
S1. L	Session2	−0.006 ± 0.193	−0.059 ± 0.162	−0.031 ± 0.256	0.020 ± 0.121	0.733
	Session3	−0.098 ± 0.223	0.068 ± 0.133^†^	0.043 ± 0.172	0.030 ± 0.081	0.044
	Session1	−0.007 ± 0.056	0.055 ± 0.164	0.029 ± 0.157	0.007 ± 0.098	0.604
S1. R	Session2	−0.001 ± 0.061	−0.122 ± 0.169^*#^	0.057 ± 0.086	−0.007 ± 0.087	<0.001
	Session3	−0.031 ± 0.220	0.026 ± 0.076^†^	0.061 ± 0.112	0.020 ± 0.077	0.354
	Session1	−0.003 ± 0.030	0.015 ± 0.128	0.012 ± 0.128	−0.012 ± 0.081	0.901
SMA&PM. L	Session2	0.027 ± 0.092	−0.034 ± 0.092^*^	0.008 ± 0.094	−0.034 ± 0.114	0.269
	Session3	−0.001 ± 0.061	0.017 ± 0.086^†^	0.030 ± 0.113	0.043 ± 0.055^*^	0.605
	Session1	0.005 ± 0.037	0.019 ± 0.124	0.051 ± 0.079	0.005 ± 0.078	0.507
SMA&PM. R	Session2	0.034 ± 0.078	−0.055 ± 0.098^*^	0.015 ± 0.060	−0.052 ± 0.179	0.073
	Session3	0.021 ± 0.100	0.008 ± 0.083^†^	0.046 ± 0.073	0.030 ± 0.052	0.613

*: *p* < 0.05 compared to session1 (baseline) within group.

†: *p* < 0.05 compared to session2 within group.

#: *p* < 0.05 compared to SH group at each time point.

*p* value: one-way ANOVA comparing four groups at each time point.

HbR analyses are reported in Supplementary Table S1. Overall, HbR effects were weaker and less spatially consistent than the primary HbO findings. No HbR omnibus effect or prespecified headline contrast survived correction for multiple comparisons.

### Functional connectivity patterns

All active acupuncture groups demonstrated enhanced prefrontal interhemispheric connectivity versus sham post-removal. Bilateral FPA connectivity showed between-group differences (*F* = 3.207, *p* = 0.031), with all active groups exhibiting stronger connectivity than SH (DS: mean difference = −0.318, *p* = 0.024; BI: −0.297, *p* = 0.036; NDS: −0.293, *p* = 0.045). Right DLPFC-FPA connectivity similarly differed (*F* = 3.189, p = 0.031), with all active groups exceeding sham (DS: −0.277, *p* = 0.027; BI: −0.266, *p* = 0.032; NDS: −0.260, *p* = 0.046).

The DS group produced dynamic frontoparietal-somatosensory connectivity. Right DLPFC to left S1 connectivity increased during retention (baseline to retention: 0.241, *p* = 0.047) and decreased post-removal (retention to post-removal: −0.237, *p* = 0.029). Right FPA to left S1 showed parallel dynamics (increase: 0.219, *p* = 0.039; decrease: −0.237, *p* = 0.020), as did left FPA to left S1 (increase: 0.219, *p* = 0.033; decrease: −0.236, *p* = 0.031).

BI selectively strengthened frontoparietal-motor connectivity post-removal. Right FPA to right M1 progressively strengthened (*F* = 3.972, *p* = 0.026; baseline to post-removal: 0.252, *p* = 0.003). Right DLPFC to right M1 similarly increased (0.226, *p* = 0.006), as did connections to left motor regions (right FPA to left M1: 0.212, *p* = 0.021; right FPA to left SMA/PM: 0.126, *p* = 0.023). Post-removal right DLPFC to left SMA/PM showed stronger BI than DS connectivity (mean difference = −0.184, *p* = 0.032).

The NDS group demonstrated delayed cross-hemispheric sensorimotor integration. Post-removal right S1 to left M1 connectivity differed between groups (*F* = 3.216, *p* = 0.031), with NDS exceeding both DS (0.334, *p* = 0.003) and BI (0.288, *p* = 0.009). Within NDS, this connectivity increased from retention to post-removal (0.212, *p* = 0.026).

**Figure 4:**
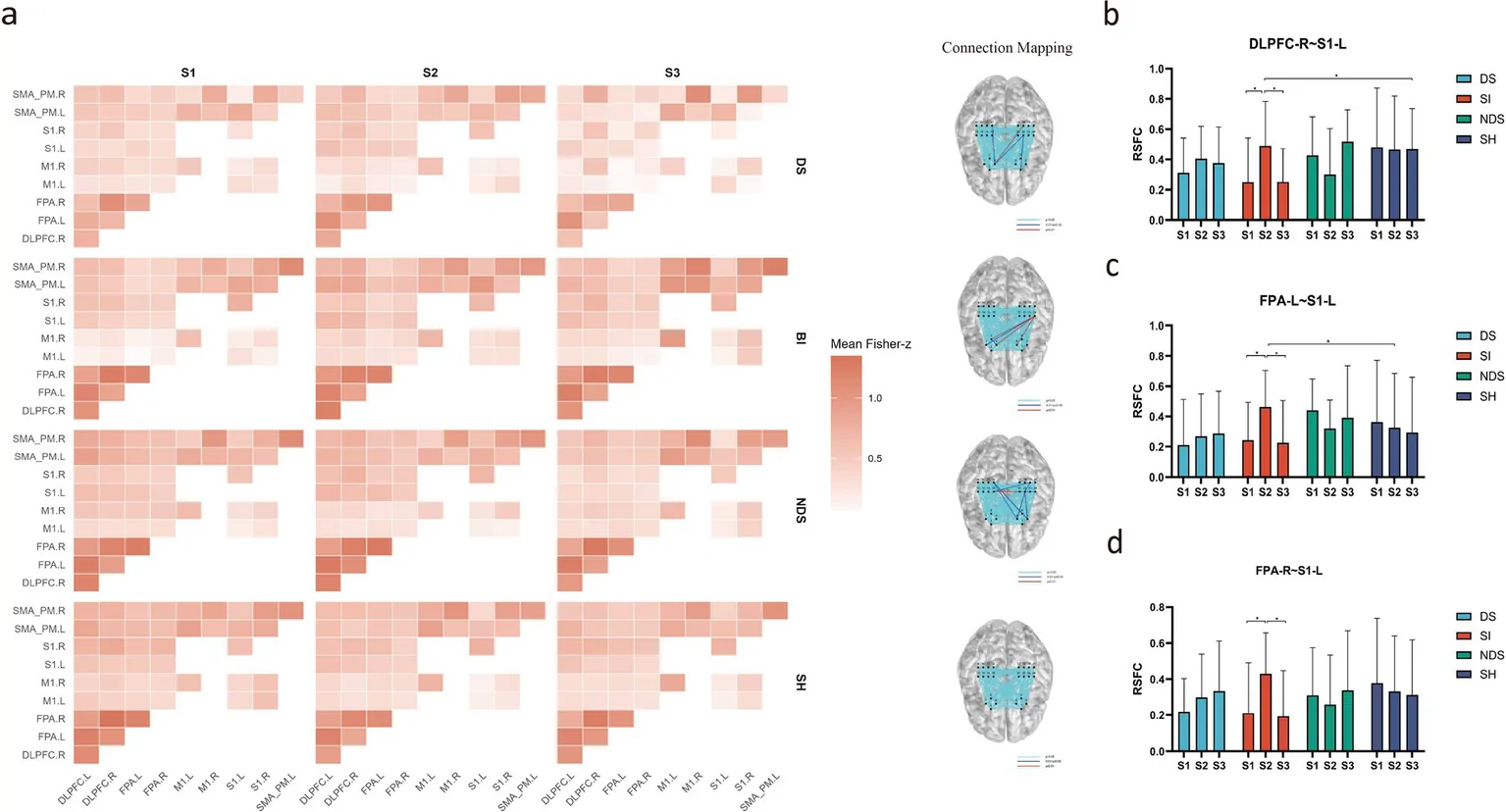
Functional connectivity changes across needling configurations. **(a)** Correlation matrices for DS, BI, NDS, and SH groups across sessions, showing inter-regional connectivity patterns among bilateral DLPFC, FPA, M1, S1, and SMA/PM regions. Brain maps illustrate thresholded connectivity patterns for each group. **(b–d)** Quantitative analysis of selected connectivity pathways: **(b)** DLPFC.R–S1.L, **(c)** FPA.L–S1.L, and **(d)** FPA.R–S1.L. Functional connectivity reflects statistical association between regional HbO time series and should not be interpreted as directed or causal neural communication. *p < 0.05, **p < 0.01.

The DS group showed consistently weaker motor connectivity post-removal, including left M1 to right SMA/PM (versus NDS: −0.323, *p* = 0.003), bilateral SMA/PM (versus SH: −0.319, *p* = 0.021), and right M1 to right SMA/PM (versus SH: −0.302, *p* = 0.037). Configuration-specific functional connectivity patterns are illustrated in Figure 4 and Supplementary Table S2.

### Small-world graph metrics indicate network reorganization

**Figure 5:**
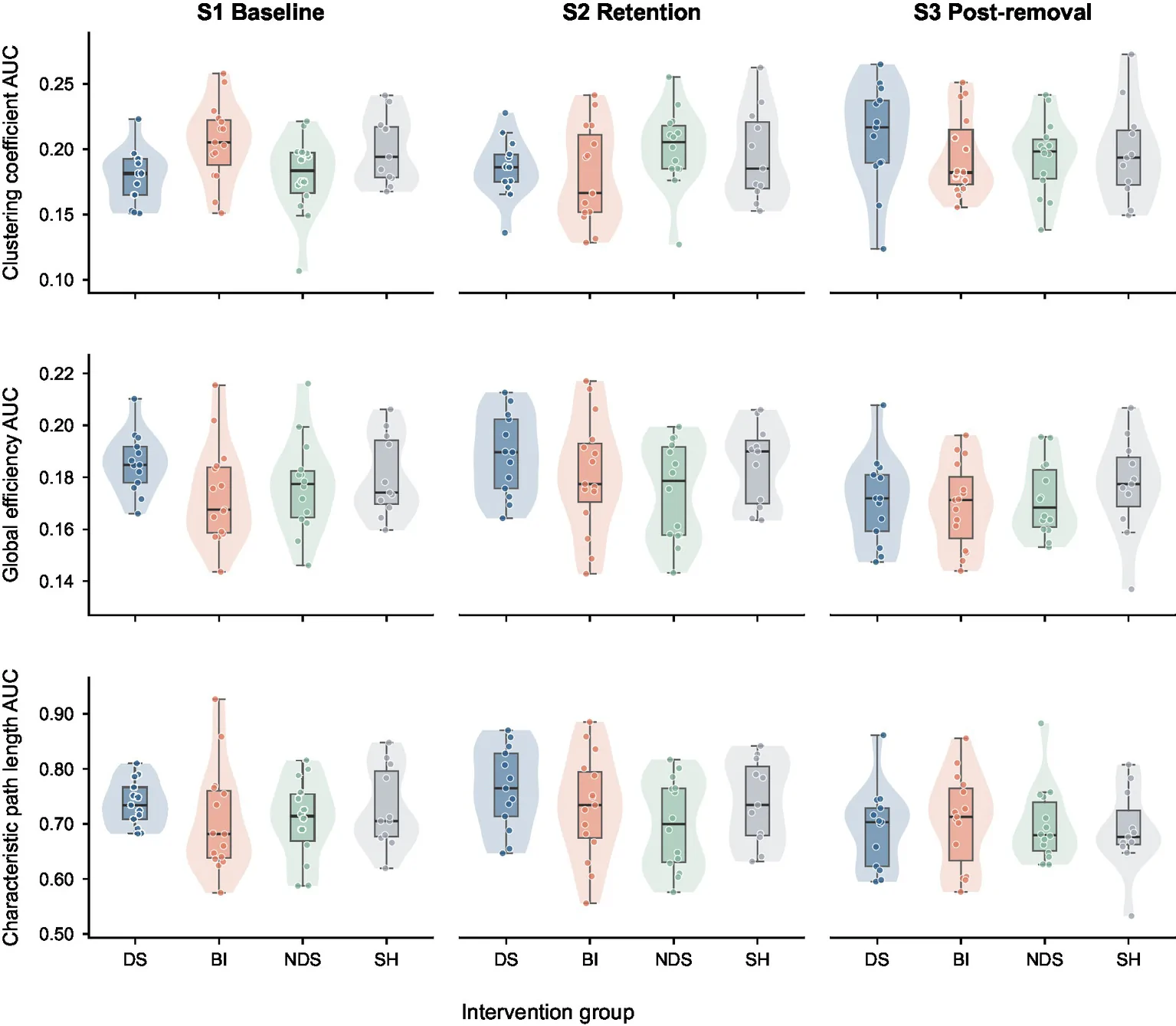
Small-world graph reorganization across sessions and intervention groups. Violin plots overlaid with box plots showing the area under the curve (AUC) of three small-world network metrics across the integration density range. Metrics include global efficiency, clustering coefficient, and characteristic path length. Group and session effects are reported with FDR correction where applicable.

Global efficiency changed significantly by session (*F* = 5.20, FDR *p* = 0.021, partial η^2^ = 0.089). Clustering coefficient showed a group-by-session interaction (*F* = 3.08, FDR *p* = 0.024, partial η^2^ = 0.148). Characteristic path length showed a near-threshold session effect (*F* = 3.48, FDR *p* = 0.051). Small-world graph metrics across sessions and groups are shown in Figure 5 and summarized in Supplementary Table S2. The graph findings show that the group differences were not limited to single ROIs or selected FC edges.

## Discussion

This study provides a systematic fNIRS comparison of cortical responses across dominant-side, non-dominant-side, bilateral, and sham LR3-LI4 stimulation in healthy right-handed adults. The main finding was that needling configuration was associated with phase-specific cortical activation and functional connectivity changes, with graph metrics providing complementary evidence of network-level reorganization. During needle retention, dominant-side stimulation was associated with greater HbO responses in the left dorsolateral prefrontal cortex (DLPFC) and frontopolar area (FPA) than bilateral and sham stimulation (17–19), suggesting stronger recruitment of prefrontal regions involved in attentional and cognitive-affective regulation. In contrast, bilateral stimulation showed attenuated prefrontal and somatosensory responses during retention followed by post-removal rebound, indicating a temporally distinct response profile rather than a simple amplification of unilateral stimulation. Non-dominant-side stimulation produced a more somatosensory-dominant pattern, with greater right primary somatosensory cortex (S1) activation than bilateral stimulation and comparatively limited prefrontal involvement. Together, these findings indicate that the same LR3-LI4 prescription produced configuration-dependent cortical response patterns that varied across retention and post-removal phases.

Bilateral stimulation is often expected to produce broader cortical engagement because homologous acupoints are stimulated on both sides of the body (2). However, the present findings do not support a simple additive cortical-response model. Compared with dominant-side stimulation, bilateral LR3-LI4 stimulation showed lower prefrontal HbO responses during retention and a delayed post-removal rebound pattern. One possible neurophysiological explanation is that simultaneous bilateral somatosensory input may increase interhemispheric interaction, including transcallosal inhibitory or redistributive processes, rather than simply summating activation across hemispheres. This interpretation is consistent with evidence that bilateral sensory stimulation can modulate cortical excitability through interhemispheric pathways (5, 6, 26), although the present fNIRS data cannot directly measure callosal inhibition. The greater prefrontal HbO response observed during dominant-side stimulation may indicate that unilateral input from the dominant limb is processed through a more focused prefrontal-sensorimotor regulatory pathway in right-handed participants. This finding suggests that laterality may be an important neurophysiological variable when interpreting cortical responses to LR3-LI4 acupuncture. Whether these cortical signatures can inform individualized acupoint selection requires validation in patient populations with clinical outcomes and longer follow-up.

The enhanced prefrontal activation with dominant-side acupuncture likely reflects hemispheric specialization patterns in right-handed individuals. In such individuals, the left hemisphere exhibits enhanced connectivity with the contralateral right-side limbs through more developed corticospinal pathways and denser thalamocortical projections (3, 4). Our findings demonstrate that dominant-side needling produced DLPFC activation greater than BI and SH during retention, aligning with the enhanced sensory processing capacity of the dominant hemisphere for contralateral limb inputs.

The bilateral acupuncture group exhibited a distinct biphasic temporal profile that differs fundamentally from unilateral patterns. During needle retention, the BI group showed significantly lower activation than the DS group in left DLPFC and bilateral FPA, accompanied by significant right primary somatosensory cortex (S1) deactivation. Following needle removal, however, the BI group demonstrated significant rebound increases in these regions, a pattern absent in unilateral groups. This biphasic response suggests that bilateral stimulation engages interhemispheric inhibition mechanisms (5). When both hemispheres receive simultaneous input, competition for processing resources and reciprocal inhibition through the corpus callosum may initially dampen activation in each hemisphere individually (26). Supporting this interpretation, Norata demonstrated interhemispheric inhibition in primary sensory cortex during bilateral median nerve stimulation (5), while Yuan found that bilateral acupuncture produced stronger motor cortex activation than unilateral approaches in stroke patients (25). The subsequent rebound likely reflects release from this inhibition, allowing delayed expression of accumulated homeostatic adjustments. This temporal dissociation between unilateral and bilateral responses indicates that these configurations engage fundamentally different neural mechanisms rather than simply varying in magnitude.

Functional connectivity analysis suggested configuration-specific patterns of statistical association among cortical regions. These findings should be interpreted as associative network measures because resting-state FC reflects covariance between regional HbO time series and does not establish directed or causal neural communication. The DS group demonstrated dynamic frontoparietal-somatosensory connectivity that increased during retention and decreased after removal. Specifically, connectivity increased between right DLPFC and left S1 and between bilateral FPA and left S1. This temporal profile is consistent with transient coupling between prefrontal regions and primary sensory cortex during dominant-side stimulation between executive control regions and primary sensory cortex receiving contralateral input from the needled dominant-side limbs, potentially enhancing salience and cognitive processing of acupuncture sensations (10, 12).

In contrast, the BI group exhibited progressive strengthening of frontoparietal-motor connectivity that continued increasing after needle removal, particularly between right FPA and bilateral primary motor cortex (M1) and between right DLPFC and bilateral M1. This sustained enhancement may reflect greater involvement of motor-related networks after bilateral stimulation rather than sensory-attentional networks. Although Ning studied GB34 acupuncture in patients with subcortical stroke rather than LR3-LI4 stimulation in healthy adults, their resting-state fMRI findings support the broader idea that acupuncture can modulate bilateral motor-network connectivity (42). The continued strengthening post-removal indicates that bilateral needling was associated with persistent motor network connectivity changes extending beyond the immediate stimulation period, as confirmed by a few researchers who demonstrated sustained neural effects following needle cessation (23, 24). Given the resting-state condition, this finding is particularly intriguing and suggests that bilateral acupuncture may prime motor systems for subsequent action or engage motor imagery processes even without explicit motor tasks.

The NDS group showed initial suppression of frontoparietal-motor connectivity during retention, particularly between left DLPFC and right M1 and between left FPA and right M1, followed by delayed enhancement of somatosensory-motor connectivity after removal, specifically from right S1 to left M1. This pattern suggests non-dominant-side stimulation may initially fail to effectively engage frontoparietal-motor integration, requiring additional time and network reconfiguration for compensatory processing. The delayed emergence of connectivity changes indicates that non-dominant-side needling follows a fundamentally different trajectory than dominant-side needling. In right-handed individuals, the dominant left hemisphere exhibits enhanced connectivity with contralateral right-side limbs, whereas the non-dominant right hemisphere has relatively weaker connectivity with contralateral left-side limbs (43, 44). Zabihhosseinian demonstrated significant differences in early somatosensory evoked potentials between dominant and non-dominant hands during motor acquisition (44), while Poole showed that preparing non-dominant hand responses involves temporarily reversing an asymmetry in excitability that normally favors the dominant hemisphere (43). Our results showing minimal immediate cortical effects during retention for the NDS group align with the reduced processing efficiency of the non-dominant hemisphere for sensory input from its contralateral limbs.

All three active groups showed stronger bilateral FPA connectivity and right DLPFC-FPA connectivity compared with sham following needle removal, indicating a common effect of genuine acupuncture on prefrontal interhemispheric integration regardless of configuration. However, configuration-specific effects emerged most prominently in sensorimotor integration patterns. Only DS and BI groups showed motor-related connectivity differences from sham, while the NDS group showed conspicuous absence of connectivity differences from sham across most networks during retention. This indicates that non-dominant-side stimulation produces minimal immediate cortical network effects in healthy right-handed individuals, although delayed effects emerged post-removal. The distinct temporal and spatial patterns across configurations challenge historical assumptions in traditional acupuncture that attribute equal immediate effects to different needle placements regardless of hemispheric lateralization (14). Graph metrics provide a broader topology-level view of these findings. Global efficiency reflects distributed network integration, whereas clustering coefficient reflects local network segregation (40). The session effect in global efficiency indicates that network integration varied across baseline, retention, and post-removal phases. The group-by-session effect in clustering coefficient indicates that local network organization varied by needling configuration and stimulation phase. These results suggest that LR3-LI4 needling configuration was associated with shifts in the balance between network integration and local specialization, consistent with small-world principles (40). However, these graph metrics remain statistical descriptors of HbO connectivity organization and should not be interpreted as evidence of directed or causal neural communication.

These configuration-dependent patterns suggest that LR3-LI4 stimulation may engage distinct cortical response profiles depending on needling side and stimulation phase. In the present healthy-adult sample, dominant-side stimulation was associated with greater retention-phase prefrontal HbO responses, whereas bilateral stimulation was associated with delayed motor-network connectivity changes and somatosensory rebound. These statistically supported neuroimaging findings suggest that different needling configurations may engage partially distinct cortical response profiles; however, they do not establish that one configuration is clinically preferable to another. Prior work has also shown that acupuncture can modulate left DLPFC activity during executive tasks in patients with severe depression (45), supporting the broader relevance of prefrontal cortical modulation in acupuncture research. In contrast to the DS-related prefrontal pattern, the BI-related delayed connectivity and rebound effects suggest a temporal profile centered more on post-stimulation sensorimotor network organization. Thus, dominant-side and bilateral LR3-LI4 stimulation appear to differ not only in response magnitude, but also in cortical timing and network emphasis. This pattern supports treating needling configuration as an important factor in acupuncture neuroimaging and as a consideration when designing stimulation protocols.

Several limitations warrant consideration. First, this study enrolled healthy right-handed adults and measured short-term cortical hemodynamic responses rather than clinical outcomes. Although several cortical activation and connectivity differences reached statistical significance, statistical significance in a short-term neuroimaging endpoint should not be equated with clinical significance. Therefore, the findings should be interpreted as neurophysiological evidence that LR3-LI4 needling configuration is associated with different cortical response patterns, not as evidence of therapeutic efficacy or clinical superiority. Whether these activation and connectivity signatures predict symptom relief, functional recovery, treatment tolerance, or response durability must be tested in patient populations. Second, the restriction to right-handed participants was appropriate for reducing heterogeneity in a dominant-side design, but it limits generalizability to left-handed or ambidextrous individuals. Because the interpretation relies partly on hemispheric specialization and limb dominance, future studies should stratify by handedness or model handedness as a biological moderator. Third, although the total sample size was supported by the *a priori* power analysis, the number of participants within each intervention group remained modest, particularly in the NDS (*n* = 14) and SH (*n* = 11) groups. This may reduce the stability of subgroup-specific activation estimates and network-level measures, including functional connectivity and graph-theoretic metrics. Larger independent cohorts are needed to confirm the reproducibility of these configuration-specific cortical and network patterns. Fourth, the findings are specific to a single LR3-LI4 intervention and may not generalize to other acupoint prescriptions, stimulation intensities, retention durations, or repeated-treatment protocols. Although LR3-LI4 is a commonly used point combination, future studies should compare different prescriptions and vary retention duration to determine whether temporal dynamics differ across needling configurations. Fifth, the weaker HbR findings should be interpreted cautiously. HbO and HbR are physiologically related but differ in signal amplitude, sensitivity to cortical blood-flow changes (36), and susceptibility to measurement noise; therefore, the absence of significant HbR effects does not necessarily negate the primary HbO activation pattern.

## Conclusion

This fNIRS study shows that LR3-LI4 needling configuration is associated with phase-specific cortical activation and network organization in healthy right-handed adults. Dominant-side stimulation showed greater retention-phase prefrontal HbO responses and transient frontoparietal-somatosensory connectivity changes; non-dominant-side stimulation showed a more somatosensory-dominant pattern, and bilateral stimulation showed attenuated retention-phase responses followed by post-removal rebound. These findings do not support a simple additive cortical-response model for bilateral needling and should be interpreted as short-term neurophysiological evidence. Future studies should test whether these configuration-dependent cortical signatures are reproducible across handedness groups and whether they predict clinically meaningful outcomes in patient populations, including those with chronic pain, anxiety or depressive symptoms, and post-stroke sensorimotor impairment.

## Data Availability

The original contributions presented in the study are included in the article/Supplementary material, further inquiries can be directed to the corresponding author.
