# Supplementary material for: LR3-LI4 needling side and configuration are associated with phase-specific cortical activation and network organization: a functional near-infrared spectroscopy study in healthy adults
Source: Front Neurol. 2026 Aug 13;17:1815369. doi: 10.3389/fneur.2026.1815369 (PMC13518510; doi:10.3389/fneur.2026.1815369)
Supplement: Supplementary file 1 [file Table_1.DOCX]

# Supplementary Table S1. HbR analysis of ROI-level activation effects

| **ROI** | **Model term** | **Numerator df** | **Denominator df** | **F** | **Raw p value** | **FDR-adjusted p value** | **Partial eta-squared** |
| --- | --- | --- | --- | --- | --- | --- | --- |
| DLPFC.L | group | 3 | 159.0 | 2.326 | 0.077 | 0.384 | 0.042 |
| DLPFC.L | session | 2 | 159.0 | 1.163 | 0.315 | 0.709 | 0.014 |
| DLPFC.L | group:session | 6 | 159.0 | 1.248 | 0.285 | 0.661 | 0.045 |
| DLPFC.R | group | 3 | 53.0 | 1.195 | 0.321 | 0.612 | 0.063 |
| DLPFC.R | session | 2 | 106.0 | 1.024 | 0.363 | 0.709 | 0.019 |
| DLPFC.R | group:session | 6 | 106.0 | 1.66 | 0.138 | 0.46 | 0.086 |
| FPA.L | group | 3 | 159.0 | 1.459 | 0.228 | 0.612 | 0.027 |
| FPA.L | session | 2 | 159.0 | 0.324 | 0.724 | 0.805 | 0.004 |
| FPA.L | group:session | 6 | 159.0 | 1.16 | 0.33 | 0.661 | 0.042 |
| FPA.R | group | 3 | 159.0 | 4.421 | 0.005 | 0.051 | 0.077 |
| FPA.R | session | 2 | 159.0 | 1.642 | 0.197 | 0.709 | 0.02 |
| FPA.R | group:session | 6 | 159.0 | 0.531 | 0.784 | 0.865 | 0.02 |
| M1.L | group | 3 | 159.0 | 0.541 | 0.655 | 0.723 | 0.01 |
| M1.L | session | 2 | 159.0 | 1.277 | 0.282 | 0.709 | 0.016 |
| M1.L | group:session | 6 | 159.0 | 2.17 | 0.049 | 0.46 | 0.076 |
| M1.R | group | 3 | 53.0 | 1.133 | 0.344 | 0.612 | 0.06 |
| M1.R | session | 2 | 106.0 | 0.862 | 0.425 | 0.709 | 0.016 |
| M1.R | group:session | 6 | 106.0 | 0.419 | 0.865 | 0.865 | 0.023 |
| S1.L | group | 3 | 53.0 | 0.777 | 0.512 | 0.723 | 0.042 |
| S1.L | session | 2 | 106.0 | 1.756 | 0.178 | 0.709 | 0.032 |
| S1.L | group:session | 6 | 106.0 | 0.588 | 0.739 | 0.865 | 0.032 |
| S1.R | group | 3 | 159.0 | 0.586 | 0.625 | 0.723 | 0.011 |
| S1.R | session | 2 | 159.0 | 0.049 | 0.952 | 0.952 | 0.001 |
| S1.R | group:session | 6 | 159.0 | 0.696 | 0.653 | 0.865 | 0.026 |
| SMA_PM.L | group | 3 | 159.0 | 0.443 | 0.723 | 0.723 | 0.008 |
| SMA_PM.L | session | 2 | 159.0 | 0.505 | 0.605 | 0.756 | 0.006 |
| SMA_PM.L | group:session | 6 | 159.0 | 1.766 | 0.109 | 0.46 | 0.062 |
| SMA_PM.R | group | 3 | 159.0 | 1.062 | 0.367 | 0.612 | 0.02 |
| SMA_PM.R | session | 2 | 159.0 | 0.687 | 0.505 | 0.721 | 0.009 |
| SMA_PM.R | group:session | 6 | 159.0 | 0.536 | 0.78 | 0.865 | 0.02 |

Note. HbR = deoxygenated hemoglobin; HbO = oxygenated hemoglobin; FDR = false discovery rate. HbR was analyzed as a secondary corroborative chromophore. Nonsignificant findings are reported for transparency. Because HbO was prespecified as the primary fNIRS endpoint, nonsignificant HbR effects were not interpreted as contradicting the primary HbO findings.

**Supplementary Table S2. Small-world graph-theoretic results**

|  | **Effect** | **Statistic** | **Raw p value** | **FDR-adjusted p value** | **Effect size** |
| --- | --- | --- | --- | --- | --- |
| Global efficiency AUC | Session effect | F = 5.199 | 0.007 | 0.021 | η²= 0.089 |
| Clustering coefficient AUC | Group x session interaction | F = 3.081 | 0.008 | 0.024 | η² = 0.148 |
| Characteristic path length AUC | Session effect | F = 3.484 | 0.034 | 0.051 | η² = 0.062 |

Abbreviations: AUC, area under the curve across network-density thresholds; FDR, false discovery rate; HbO, oxygenated hemoglobin; ROI, region of interest. FDR correction was applied within the graph-metric analysis family. Characteristic path length is reported as near-threshold after FDR correction.
